# Whole-Body Cryostimulation as an Adjunctive Treatment for Neurophysiologic Tinnitus and Associated Disorders: Preliminary Evidence from a Case Study

**DOI:** 10.3390/jcm13040993

**Published:** 2024-02-08

**Authors:** Paolo Piterà, Riccardo Cremascoli, Angelo Alito, Laura Bianchi, Federica Galli, Federica Verme, Jacopo Maria Fontana, Matteo Bigoni, Lorenzo Priano, Alessandro Mauro, Paolo Capodaglio

**Affiliations:** 1 Research Laboratory in Biomechanics, Rehabilitation and Ergonomics, IRCCS Istituto Auxologico Italiano, San Giuseppe Hospital, 28824 Verbania, Italy; f.verme@auxologico.it (F.V.); j.fontana@auxologico.it (J.M.F.); p.capodaglio@auxologico.it (P.C.); 2 Unit of Neurophysiology, IRCCS Istituto Auxologico Italiano, San Giuseppe Hospital, 28824 Verbania, Italy; r.cremascoli@auxologico.it (R.C.); laurabianchitnfp@gmail.com (L.B.); gallif.tnfp@gmail.com (F.G.); m.bigoni@auxologico.it (M.B.); lorenzo.priano@unito.it (L.P.); alessandro.mauro@unito.it (A.M.); 3 Department of Biomedical, Dental Sciences and Morphological and Functional Images, University of Messina, 98122 Messina, Italy; alitoa@unime.it; 4 Department of Neurosciences “Rita Levi Montalcini”, University of Turin, 10124 Torino, Italy; 5 Department of Surgical Sciences, University of Torino, Physical Medicine and Rehabilitation, 10121 Torino, Italy

**Keywords:** neurophysiologic tinnitus and associated disorders, whole-body cryostimulation, inflammation, rehabilitation

## Abstract

Background: Tinnitus, which is often associated with reduced quality of life, depression, and sleep disturbances, lacks a definitive treatment targeting its pathophysiological mechanism. Inflammatory markers like TNF-α have been linked to tinnitus, thereby underlining the necessity for innovative therapies. This case study investigates the potential benefits of a multi-approach rehabilitation intervention involving whole-body cryostimulation (WBC) for a 47-year-old male suffering from chronic neurophysiologic tinnitus, who had underwent various unsuccessful treatments from 2005. Methods: the patient underwent a personalized, multidisciplinary rehabilitation intervention covering diet, pharmacotherapy, physiotherapy and physical activity classes tailored to the patient’s needs and capacities, repetitive transcranial magnetic stimulation (rTMS), and whole-body cryostimulation (WBC). Results: The adjunctive WBC intervention resulted in a significant progressive improvement in tinnitus severity (tinnitus handicap inventory Δ% = −46.3%, VAS tinnitus score Δ% = −40%). Additional positive outcomes were noted in sleep quality (PSQI Δ% = −41.67%), emotional wellbeing (BDI Δ% = −41.2%), and quality of life (SF-36, WHO-5 Δ% = +16.5). Conclusions: This study supports the existing literature suggesting the potential of WBC as an adjunct in a multi-approach intervention in ameliorating tinnitus severity and tinnitus-associated disorders. However, randomized controlled trials in larger populations, which specifically consider WBC’s effects on tinnitus, are necessary to confirm these findings and to explore the mechanisms that underlie the observed improvements.

## 1. Introduction

Tinnitus (from the Latin word “tinnire” = ring) is synonymous with ear noise or ear ringing and corresponds to the perception of an acoustic phenomenon that is not caused by external impulses. It is generated at a certain point in the auditory system, mostly in the cochlea, and then it is processed and perceived as noise annoyance in the cortex. 

Certain investigators distinguish between somatic tinnitus and neurophysiologic tinnitus [1]. In this classification, somatic tinnitus is categorized as tinnitus with an underlying medical condition that creates internal acoustic mechanical sounds (vascular, muscular, respiratory, or temporo-mandibular joint origin). Somatic tinnitus can be cured by identifying the source or the underlying condition and appropriately treating it [2,3]. On the other hand, neurophysiologic tinnitus, in which the “phantom sounds” are attributed to an impairment in the neurological auditory pathway, is the most common diagnosis. This form of tinnitus can only be heard by the patient and cannot be directly observed by a physician, thus making it difficult to evaluate [3]. 

Epidemiologic studies of Europe and the United States of America predict that about a quarter of all people have experienced a tinnitus sensation at least once, while 10–15% experience tinnitus over a longer period. Currently, only 3–5% are considered to be in need of treatment, and half of them suffer significantly [1,4]. 

Quality of life is negatively affected by tinnitus in 1–4% of the general population, thereby making tinnitus a substantial medical and socio-economic problem [4]. Numerous studies have revealed close associations between mental illnesses and tinnitus, and significantly higher rates of comorbid anxiety, depression, panic, low self-esteem, and sleep disorders have been reported for tinnitus sufferers [5]. 

Additionally, tinnitus-associated dysfunctional cognition, which includes catastrophic thinking and avoidance cognitions, is strongly correlated with measures of tinnitus distress. The majority of the studies found that depression either predisposes someone to tinnitus or occurs as a consequence of tinnitus [6]. Furthermore, sleep disturbance and annoyance are among the problems more frequently reported by individuals with chronic tinnitus, and increased sleep disturbance has been shown to closely correlate with increased tinnitus severity, thus providing support for the hyperactive limbic and sympathetic system model of tinnitus intolerance [7]. 

For patients reporting bothersome and intolerable tinnitus and seeking treatment, it has been suggested that the occurrence of tinnitus may produce an inappropriate activation of the limbic and sympathetic components of the autonomic nervous system [5,8]. Knowledge of the pathophysiology of tinnitus is essential for the development of new treatment strategies. 

Previous research has established that tinnitus is associated with aberrant neuronal firing regardless of the cause of tinnitus [9,10]. The pathophysiology of tinnitus is closely related to that of acquired hearing loss, and there is increasing evidence that inflammation may contribute to the process of hearing loss. Accordingly, neuroinflammation may be one of the key processes in the development of tinnitus [11,12]. 

Current treatments for tinnitus, such as hearing aids, cognitive behavioral therapy, and sound therapy, aim to reduce tinnitus perception or to develop new coping strategies [4]. However, there is no treatment that targets the pathophysiologic mechanism. Several animal studies consistently demonstrate the central role of TNF-α in the development and persistence of tinnitus, with an elevated TNF-α level observed after tinnitus induction and its infusion causing tinnitus in healthy mice [12]. Despite the importance of TNF-α, elevated levels of IL-6 and IL-10 observed for chronic tinnitus suggest a broader inflammatory response [13]. Targeting neuroinflammatory markers is emerging as a potential therapeutic choice, particularly in the acute phase of tinnitus, while their role in the chronic phase remains less defined [4]. 

Whole-body cryostimulation (WBC) is a medical physical treatment that involves the whole body being exposed to extremely low temperatures of up to −110/−140 °C for 2–3 min. It is well known in the sports world for its proven analgesic and anti-inflammatory properties [14], and WBC is attracting increasing clinical interest. Numerous studies, in fact, have unveiled favorable outcomes for individuals dealing with chronic pain, fatigue, and conditions associated with inflammation, such as neurological disorders, fibromyalgia, and obesity [15,16,17,18].

WBC has been shown to modulate the autonomic nervous system, elicit endorphin release from the brain, and exert molecular and systemic anti-inflammatory effects that are similar to those induced through exercise [19]. Furthermore, WBC has been found to exert beneficial effects on depression, mood, and quality of life, as well as improving sleep and reducing the severity of insomnia [20,21,22]. 

These findings suggest that WBC could serve as a supplementary treatment for tinnitus sufferers by not only restoring nervous system homeostasis but also reducing systemic inflammation and ameliorating tinnitus-associated conditions. To the best of our knowledge, there is only one previous study, which was conducted in Poland, on the effects of WBC on tinnitus. It showed that WBC was effective in reducing the severity of tinnitus for the majority of the study participants and, in some cases, resulted in complete resolution [23]. The purpose of this work is to provide additional evidence of the potentially positive effects of a multi-approach series of rehabilitation interventions that includes a WBC cycle in the treatment of neurophysiologic tinnitus while also evaluating tinnitus-associated conditions, such as overall quality of life, sleep quality, and emotional wellbeing.

## 2. Materials and Methods

### 2.1. Case Description

A 47-year-old male patient was admitted to the Neurorehabilitation Unit of Piancavallo Hospital to undergo a rehabilitation program for insomnia, obesity, and obstructive sleep apnea syndrome (OSAS). The patient suffered from class 1 obesity (BMI 32), which had developed following steroid therapy for SARS-CoV-2 infections that had initially taken place in July 2021 and then in April 2023. In May 2023, a diagnosis of severe OSAS was established, which is currently being managed through nocturnal continuous positive airway pressure (CPAP); following the OSAS diagnosis, the patient was strongly recommended to start a weight loss program. The patient reported mixed insomnia (difficulty in falling asleep even with Delorazepam and melatonin supplementation) and recurrent nocturnal awakenings along with daytime hypersomnia, difficulty in concentration, and memory disturbances. Additionally, the patient had been suffering an anxious–depressive syndrome for 20 years, which was well controlled with Duloxetine at the time of admission. The patient also presented psoriasis that was topically treated with corticosteroid therapy in cycles, irritable bowel syndrome (biopsy revealing lymphocytic colitis), and hepatic steatosis. Moreover, the patient had been experiencing chronic tinnitus for nearly 20 years.

### 2.2. Tinnitus History and Description 

The patient’s experience with tinnitus started in 2005 subsequent to cervical chiropractic sessions; it was initially localized in the right ear and has persisted to the present day. The auditory perception manifests itself as a waterfall’s rushing sound, predominantly in the right ear but radiating into the left one too. The patient frequently reports a sense of muffled hearing and recurrent otitis in the right ear. Notably, during airplane landings, he experiences ear plugging sensations that are accompanied by acute pain. The patient’s tinnitus has been profoundly debilitating, leading to severe discomfort. In fact, he reports a substantial negative impact on overall quality of life, social wellbeing, and occupational performance, necessitating sick leave. Interestingly, while the tinnitus itself does not induce insomnia in this patient, it exacerbates the existing insomnia caused by severe OSAS, thereby contributing to the patient’s sleep disturbances. Over the years, various interventions had been attempted, including corticosteroid medications, tinnitus retraining therapy (TRT) [24] involving ear-inserted devices, repetitive transcranial magnetic stimulation (rTMS) [25] with deep stimulation over 20 sessions in 2022 and repeated in 2023, and laser therapy to the ears with no discernible benefit over 10 sessions in 2017. RTMS treatment was effective in reducing depressive symptoms but had no notable effect on the tinnitus. Cervical physiotherapy employing diverse approaches and treatments was pursued in an effort to resolve the tinnitus, albeit without success. A cerebral MRI with angiography that was conducted in March 2018 excluded the presence of anatomical lesions in the auditory pathway.

### 2.3. Symptom Assessment

Tinnitus presence and severity were evaluated through the tinnitus handicap inventory (THI) [26] before and after the WBC cycle, and to assess WBC’s effects on tinnitus severity and the progression of the symptomatology over time the patient was asked to complete a VAS (visual analogue scale) for tinnitus intensity [27] before and after each WBC session. Health-related quality of life was evaluated using the short form health survey 36 (SF-36) [28]. Sleep quality was assessed using the Pittsburgh sleep quality index (PSQI) [29]. General, subjective wellbeing was measured using the 5-item World Health Organization well-being index (WHO-5) [30]. Severity of depression was determined using Beck’s depression inventory (BDI) [31] and anxiety was assessed with Beck’s anxiety inventory (BAI) [32]. Furthermore, to ascertain whether the tinnitus could be attributed to anatomical or functional abnormalities within the auditory pathway, brainstem auditory evoked potentials (BAEPs) [33] were conducted before the initial cryostimulation session and repeated at the conclusion of the rehabilitation program. 

BAEPs were recorded using an evoked potential recording system (Synergy 12.2, CareFusion Teca Mendelec, UK). To elicit and record BAEPs, an auditory stimulus was delivered to the patient through headphones. Brainstem auditory evoked potentials were recorded using the 10:20 system (International Federation of Societies for EEG and Clinical Neurophysiology). Two channels were recorded with the active electrodes placed over the skin region of the mastoid process, which were both referenced to the vertex electrode (Cz). Waveforms from the ipsilateral and contralateral pathways were recorded simultaneously, thereby allowing for the easier recognition of individual peaks. Low- and high-frequency filters were set to frequencies between 10 and 3000 Hz, respectively. While one ear was stimulated with clicks, the other ear was masked with white noise, and an equal mixture of all frequencies within the range of human hearing (typically 20 Hz–s20 kHz) was used. To ensure maximum and consistent BAEP detection, the stimulus intensity was set to a level of 65–75 dB above the standard thresholds for the sensation level (dBSL) or the hearing level (dBHL).

The results yielded normal outcomes, suggesting the absence of anatomical or functional alterations in the patient’s auditory pathway. Therefore, tinnitus in this patient is determined to be of neurophysiological origin. Results from tests, scales, and questionnaires are shown in Table 1, Table 2 and Table 3.

### 2.4. Intervention

#### 2.4.1. Diet, Physiotherapy, and Physical Activity

In order to achieve a suitable amount of weight loss to improve the symptoms of obstructive sleep apnea syndrome (OSAS) and other obesity-related conditions, a dietary plan was established as follows:

- Total caloric intake: 1600 kcal.
- Nutritional composition: proteins 86 g (21%), lipids 50 g (28%), carbohydrates 209 g (51%).

Personalized daily physical activity and physiotherapy programs were implemented according to the patient’s capacity and needs.

#### 2.4.2. Drug Therapy and CPAP

During hospitalization, the patient was treated with the following medication:

- Esomeprazole 20 mg;
- Duloxetine 60 mg, twice daily;
- Melatonin Fast, 10 drops at 11:00 PM;
- Clobesol 0.05% cream, applied once in the morning and once in the evening.

Furthermore, nocturnal ventilatory therapy readjustment was performed using a CPAP Airsense 10 Elite, set at 10 cm H_2_O, with a nasal mask.

#### 2.4.3. Repetitive Transcranial Magnetic Stimulation (rTMS)

Before starting the WBC cycle, 10 sessions of repetitive transcranial magnetic stimulation were administered to reduce tinnitus severity, with one session carried out per day for 10 days. Specifically, an inhibitory treatment was applied to the right temporal area at a frequency of 1 Hz, with a total of 1200 stimuli. To evaluate any potential improvement in tinnitus, the tinnitus handicap inventory (THI) questionnaire was administered, which gave a score of 84 before the onset of the first repetitive transcranial magnetic stimulation (rTMS) treatment and 82 following the completion of the rTMS cycle. These results indicate no significant reduction in tinnitus, which is consistent with previous treatments using this approach. In fact, this patient had undergone several cycles of rTMS over the years but did not experience any noticeable effects on tinnitus severity.

#### 2.4.4. Whole-Body Cryostimulation (WBC)

Whole-body cryostimulation intervention consisted of a total of 10 WBC sessions performed twice a day (at 9 am and 12 pm) from 31 October to 7 November 2023. After confirming the absence of contraindications to WBC treatment according to Bad Voslau’s list of contraindications [15], the patient underwent an initial 1-min/−110 °C familiarization session during which he entered the cryochamber (Arctic, CryoScience, Rome, Italy, nitrogenous-cooling cryochamber) dressing in accordance with the rules applicable during WBC procedures as follows: minimal clothing and protection for the body’s extremities. He then underwent 10 WBC sessions that were 2 min long and conducted at −110 °C. Each treatment was performed and monitored by specially trained personnel.

**Figure 1:**
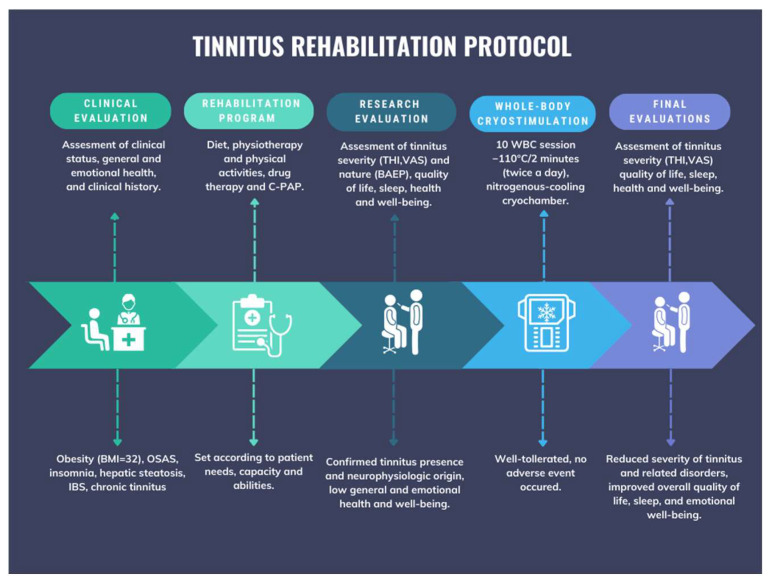
Schematic representation of the rehabilitation protocol.

A graphical representation of the intervention is provided in Figure 1.

## 3. Results

At the time of examination, the tinnitus was assessed using the Italian-validated version of the THI and was scored at 82/96, thereby confirming the presence of tinnitus.

Brainstem auditory evoked potentials (BAEPs) were conducted before the initial cryostimulation session and repeated at the conclusion of the rehabilitation program. The results showed the absence of anatomical or functional alterations in the patient’s auditory pathway, thereby confirming the neurophysiologic origin of the patient’s tinnitus. The results from the BAEPs are depicted in Table 3.

The results from all the scales and questionnaires administered are shown in Table 1 as pre-WBC, post-WBC, and range values.

The VAS tinnitus scores (Table 2) were registered before (pre-WBC) and after (post-WBC) every WBC session.

## 4. Discussion

This case report on the effects of WBC on tinnitus and related disorders describes positive outcomes in terms of the tinnitus severity, overall quality of life, sleep quality, and emotional wellbeing for a 47-year-old male admitted to our institution for a rehabilitative program targeting comorbid insomnia associated with OSAS. 

The patient had been experiencing chronic tinnitus since 2005, undergoing various treatments over the years, including pharmacotherapy, repetitive transcranial magnetic stimulation (rTMS), tinnitus retraining therapy (TRT), ear laser therapy, and several approaches and different treatments for cervical physiotherapy without noticeable improvements in tinnitus severity and its related disorders. In this study, following a rehabilitative program including a 10-session WBC cycle at −110 °C/2 min, we observed a positive impact of WBC on tinnitus severity, which was evaluated through the THI and VAS (registered before and after each WBC treatment). 

The effect became evident early in the treatment and intensified and persisted throughout the entire WBC cycle, thereby indicating rapid action on this condition (Table 2). Moreover, the patient reported unprecedented benefits compared with prior treatments, expressing an improvement in general mood, sleep, wellbeing, and quality of life. Numerous treatments are currently available for tinnitus, including drug therapy, sound-based and psychological interventions, magnetic or electrical stimulation, manual physical therapy, relaxation therapy, and complementary and alternative medicine therapies. However, these approaches generally show low efficacy, and the patient population experiences frustration and impatience as a result of this [34]. 

According to the current literature, tinnitus requires treatment mainly when psychosomatic tinnitus-related disorders, such as sleep and concentration disorders, as well as anxiety and episodes of depression occur [1]. In fact, these side effects lead the patient to have an urgent need of treatment even when tinnitus has always been considered to be the triggering factor and the patient expects palliation for the symptoms. This statement suggests that therapeutic interventions for tinnitus, when used as stand-alone treatments, are rarely effective in producing positive results [1]. 

Consequently, it could be worth investigating the effects of WBC on individuals suffering from tinnitus and related disorders since WBC has been proven to improve most tinnitus-related disorders, such as insomnia, depressive, and anxiety syndromes, that often decrease the overall quality of life of tinnitus sufferers [5,20,22,35].

This treatment may have the potential to alleviate the severity of tinnitus by mitigating systemic inflammation and restoring nervous system homeostasis. These effects could positively impact the auditory neurological pathway. However, research on the underlying potential and the effective biological mechanisms is currently lacking. 

The existing literature includes an article that investigates the effects of WBC on tinnitus patients, which showed that a cycle of WBC can decrease the severity of tinnitus and, in some cases, even resolve it [23]. 

Interestingly, studies have indicated the role of inflammation in the onset and persistence of tinnitus, particularly observing elevated TNF-α levels for individuals with tinnitus [4,13]. 

Since WBC is known to reduce inflammatory states and restore the homeostasis of the nervous system, our hypothesis was that a WBC cycle could serve as an adjuvant and complementary treatment to resolve or at least ameliorate neurophysiologic tinnitus and its related disorders. 

The results presented in this study, although promising, clearly require further randomized and controlled studies with a larger population to confirm the benefits achieved through the introduction of cryostimulation and the underlying biological mechanism of action of WBC for tinnitus. 

The patient’s positive emotional response to the treatment and satisfaction with a brief, well-tolerated intervention that was devoid of adverse effects may have contributed to the observed functional improvements, and a placebo effect cannot be entirely discounted.

We are aware that the improvement in tinnitus and the related conditions cannot be attributed solely to WBC per se as the patient underwent a series of rehabilitation interventions during hospitalization. However, considering that the previous interventions performed on this patient over the 20 years prior had not resulted in significant improvement, the benefits obtained by introducing WBC into a multi-approach rehabilitation intervention suggest its possible positive effect on tinnitus improvement. Therefore, there may be a rationale behind recommending this treatment to tinnitus sufferers who have not experienced improvement through conventional therapies. A follow-up would be necessary to study and assess the extent of improvement and its maintenance over time.

## 5. Conclusions

WBC showed promising results and should be considered as an adjuvant and complementary treatment for chronic neurophysiologic tinnitus and associated disorders. Further randomized controlled studies with larger cohorts are imperative in order to validate the observed benefits and elucidate the specific mechanisms of action of WBC in treating tinnitus.

## Figures and Tables

**Table 1:** Questionnaires and scales administered before and after the WBC cycle.

Outcome and Range	Pre	Post	Δ%
THI (0–96)	82	44	−46.3%
SF-36			
*Physical functioning*	61.5	65	+4.5%
*Role limitations due to physical health*	100	100	0
*Role limitations due to emotional problems*	100	100	0
*Energy/fatigue*	30	35	+5%
*Emotional well-being*	28	40	+12%
*Social functioning*	0	12.5	+12.5%
*Pain*	20	42.5	+22.5%
*General health*	5	15	+10%
WHO-5 (0–100)	24%	40%	+16%
PSQI (0–21)	12	7	−41.67%
BDI (0–63)	17	10	−41.2%
BAI	3	3	0

BAI= Beck’s anxiety inventory; BDI= Beck’s depression inventory; PSQI = Pittsburgh sleep quality index; SF-36 = short form health survey 36; THI= tinnitus handicap inventory; WHO-5 = 5-item World Health Organization well-being index.

**Table 2:** VAS tinnitus intensity scores before (pre-WBC) and after (post-WBC) each WBC session.

WBC Session	T1	T2	T3	T4	T5	T6	T7	T8	T9	T10
VAS tinnitus score pre-WBC	70	60	50	50	35	35	35	35	35	35
VAS tinnitus score post-WBC	70	60	45	45	35	35	30	35	35	30

WBC = Whole Body Cryostimulation; VAS = visual analog scale; T = treatment.

**Table 3:** Brainstem auditory evoked potentials (BAEPs) recorded for the right and left ears.

	Aud.Stim. dB	I Latency (ms)	II Latency (ms)	III Latency (ms)	IV Latency (ms)	V Latency (ms)	I-V Latency (ms)
ipsilateral	130 SPL	0.98	1.76	2.78	3.98	5.40	4.43
ipsilateral	125 SPL	0.94	1.66	2.80	3.98	5.12	4.18
ipsilateral	110 SPL	1.62	2.74	3.94	5	5.62	4
ipsilateral	110 SPL	1.68	2.54	4.08	5.10	5.90	4.22

## Data Availability

The data presented in this study are available within the article.
